# Ponce De Leon Spring

**Published:** 1872-07

**Authors:** 


### PONCE DE LEON SPRING.

The wonderful reputed medicinal qualities of the water of this spring have been heralded throughout the State of Georgia—and, in fact, throughout the South. Cures have been effected, it is said, by it, so surprising as would seem to at once place it in the front rank of the most celebrated mineral springs of the world. Chalybeate springs abound throughout the South, whose waters are justly renowned as of the utmost value. But the water of Ponce de Leon is not entirely chalybeate. It has not yet been submitted to analysis, but judging from its reported effects on the human system, and the cures it has been said to have performed, it is alkaline, or belongs to the class of Alkaloid-Carbonates—its special properties being alterative, tonic, diuretic and aperient.

These properties would seem to suggest it as applicable to diseases of the kidneys, bladder, etc.; and cures of these and kindred diseases of the urinary passages are reported by the most reliable citizens of Atlanta in patients having, hitherto, defied the therapeutics of our ablest physicians. It seems also peculiarly adapted to all forms of dyspepsia and rheumatic affections—many persons suffering from these ailments having, it is affirmed, been permanently benefitted and relieved.

Above and below Ponce de Leon Spring, proper,—within a few hundred yards—are strong chalybeate springs—as fine, perhaps, as are to be found in any region. These, with the peculiar alterative, tonic, diuretic and aperient qualities of Ponce de Leon, are destined, doubtless, to make the waters in the immediate vicinity of this spring the most celebrated of the South. Atlanta seems to be peculiarly fortunate. Aside from her surprising commercial growth and geographical position to the Southern States, she has a climate unequalled in salubrity and purity, which alone entitles her to the esteem of invalids in quest of health. But when the great value of her mineral waters, which surround it on every side, are considered, in connection with the facts above, she is surely destined to become, at no distant day, one of the first watering places of the country.

